# The Pattern of Dyslipidemia in Chronic Liver Disease Patients

**DOI:** 10.7759/cureus.13259

**Published:** 2021-02-10

**Authors:** Umar Farooque, Ashok Kumar Lohano, Quratulain Dahri, Nazia Arain, FNU Farukhuddin, Chinmay Khadke, Febin Prince, Rizwan Farooque, Mostafa A Shehata, Muhammad Daim Bin Zafar

**Affiliations:** 1 Neurology, Dow University of Health Sciences, Karachi, PAK; 2 Medicine, Peoples University of Medical and Health Sciences for Women, Nawabshah, PAK; 3 Internal Medicine, Peoples University of Medical and Health Sciences for Women, Nawabshah, PAK; 4 Neurology, University Hospital Cleveland Medical Center, Cleveland, USA; 5 Internal Medicine, Rural Medical College, Pravara Institute of Medical Sciences, Loni, IND; 6 Emergency Medicine, Medical University of Lublin, Lublin, POL; 7 Internal Medicine, Sindh Medical College, Karachi, PAK; 8 Medicine and Surgery, Alexandria Faculty of Medicine, Alexandria, EGY; 9 Internal Medicine, Dow University of Health Sciences, Civil Hospital Karachi, Karachi, PAK

**Keywords:** child-pugh classification, chronic liver disease, lipid profile, dyslipidemia, severity of disease, management, monitoring, risk stratification, cld, liver cirrhosis

## Abstract

Introduction

Patients with chronic liver disease are expected to report derangements in serum lipid profiles. Lipid profile monitoring is not a part of the routine management of these patients in our hospital. Few recent studies show how lipid profile varies with the severity of disease and should be considered in the management planning of such patients. The objective of this study was to determine the pattern of dyslipidemia in chronic liver disease patients.

Materials and methods

A cross-sectional study was conducted involving 171 patients of all genders aged between 18 years and 60 years presenting with chronic liver disease with disease severity graded on Child-Pugh class as A, B, and C. Lipid profile was acquired in all these patients and was compared across various subgroups. Individual serum lipid parameters were graded as normal, high, or very high. Each patient was required to provide written informed consent. Statistical Package for Social Sciences (SPSS) version 21.0 (IBM Corp. Armonk, NY) was used to analyze data statistically, taking a p-value of ≤0.05 as significant.

Results

The mean age of patients was 51.2±7.3 years. The male to female ratio came out to be 1.5:1, with 103 (60.2%) male and 68 (39.8%) female patients included in the study. The disease was classified as Child-Pugh A in 20 (11.7%) patients, Child-Pugh B in 67 (39.2%) patients, and Child-Pugh C in 84 (49.1%) patients. Forty-four (25.7%) patients were hypertensive while 62 (36.3%) were diabetic. The mean body mass index (BMI) of these patients was 25.9±2.4 kg/m^2^. Mean serum values among Child-Pugh A, Child-Pugh B, and Child-Pugh C of low-density lipoproteins (LDL) (113.15±14.08 vs. 95.58±14.25 vs. 53.46±5.90 mg/dl; p-value 0.001), high-density lipoproteins (HDL) (50.60±3.19 vs. 40.70±2.95 vs. 35.40±3.88 mg/dl; p-value 0.001), total cholesterol (174.20±17.33 vs. 164.00±17.82 vs. 128.64±24.73 mg/dl; p-value 0.001), and triglycerides (127.15±8.98 vs. 100.84±27.12 vs. 93.36±25.56 mg/dl; p-value 0.001) decreased significantly with increasing severity of disease. Nineteen (11.1%) patients had hyperlipidemia (serum values of two or more parameters above normal) while 152 (88.9%) patients had normal lipid profile. When stratified, no statistically significant difference was found in the frequency of hyperlipidemia across various subgroups based on the patient's gender, age, duration, and severity of the disease, BMI, or diabetic and hypertensive status.

Conclusions

A substantial proportion of patients with chronic liver disease had hyperlipidemia which varied with the severity of disease on Child-Pugh classification. Routine monitoring of the lipid profile of such patients is necessary for timely identification and management of dyslipidemia to improve the outcome of such patients. It also suggests an important role of lipid profile in the risk stratification and treatment of chronic liver disease patients and warrants further studies in this regard.

## Introduction

Several hepatic disorders in which normal liver tissue is replaced by fibrotic tissue are collectively termed chronic liver disease (CLD) [1]. Several metabolic abnormalities are common in CLD like dyslipidemias, clotting factor deficiencies, and in some cases venous thromboembolism due to deficiency of anticoagulation factors [2]. Dyslipidemia is a metabolic anomaly that causes an imbalance in lipids or lipoproteins [3]. Fat-soluble vitamins, dietary cholesterol, and long-chain fatty acids are absorbed and transported by lipoproteins. Lipids are present in the cell membrane and are important regulators of cellular function and the internal body environment. Exogenous and endogenous lipid metabolism cycles are controlled by the liver. The liver is also responsible for the formation of apolipoproteins, which activate enzymes responsible for lipoprotein metabolism and attachment to their specific receptors [4].

Imbalance in serum lipoprotein can be due to cirrhosis of the liver, where cholesterol of low-density lipoprotein (LDL) variant and high-density lipoprotein (HDL) variant are reduced [5]. Dyslipidemia reported in patients of CLD is remarkable in contrast with other causes of secondary dyslipidemia as the composition, appearance, serum levels, and mobility of lipoproteins is disturbed [3,6]. Ress and Kaser have reported that CLD interferes with the metabolism of lipid by disrupting the functionality of the liver, resulting in the accumulation of lipid drops in hepatocytes. Abnormal secretion of very-low-density lipoprotein (VLDL) leads to hepatic steatosis [7].

A previous study reported that the total cholesterol was markedly decreased in 15% of patients. Overall, 82.5% of patients presented with hypocholesterolemia, and 2.5% of patients presented with hypercholesterolemia. Whereas, 63.13% of patients reported low triglyceride levels, 88.13% of patients reported decreased LDL, and increased levels were reported in 4.38% of patients [4]. In another study, 69.09% of patients had decreased total cholesterol, hypercholesterolemia was not reported, and 12.72% of patients had low serum triglyceride [8].

As variation in results is mentioned in the studies above, the present study is conducted to see the pattern of dyslipidemia and lipid metabolism derangement in CLD patients and to have an idea of local prevalence. This study will help the patients and healthcare providers by early identification of the pattern of dyslipidemia and taking steps to improve the survival and outcomes of the patients.

## Materials and methods

This cross-sectional study was done for six months from August 2019 to January 2020 at the Department of General Medicine, Peoples Medical College, and Hospital, Nawabshah, Pakistan.

Sample size, inclusion, and exclusion criteria

A sample size of 171 was calculated using the World Health Organization (WHO) software, taking the prevalence of low serum triglyceride levels in patients 12.72%, the margin of error 5%, and confidence level 95%. Patients suffering from the CLD aged between 18 years and 60 years, confirmed by history and medical record of either gender were enrolled in the study. Whereas, the patients undergoing concomitant illnesses like heart failure, renal failure, or a thyroid problem, patients taking lipid-lowering drugs, patients consuming alcohol, or patients with a body mass index (BMI) of >30 kg/m^2^ were excluded from the study sample.

Sampling technique and data collection

Non-probability consecutive sampling technique was employed to sample patients. The study was conducted after approval from the ethical committee of the institute. Informed consent was also taken from all the patients enrolled before including them in the study. After taking a complete history and physical examination, a lipid profile was done to investigate the pattern of dyslipidemia. The data was entered in a predesigned proforma. Confounding variables and bias were controlled by strictly following inclusion criteria.

Statistical analysis

Statistical Package for Social Sciences (SPSS) version 21.0 (IBM, Armonk, NY) was used to analyze the collected data. Continuous variables, such as age, BMI, duration of liver disease, LDL, total cholesterol, HDL, and triglycerides, were presented as mean±SD. Serum levels of triglycerides, LDL, total cholesterol, and HDL have been compared across various subgroups using independent sample t-test/ANOVA. A p-value of ≤0.05 was considered significant. Categorical variables like gender, the severity of liver disease (Child-Pugh class), diabetes mellitus, hypertension, and pattern of dyslipidemia were presented using percentages and frequencies. Age, gender, BMI, duration, the severity of the disease, diabetes mellitus, and hypertension were stratified to address the effect of modifiers. Chi-square test was applied to assess categorical variables, taking a p-value of ≤0.05 as significant.

## Results

The mean age of the patients enrolled was 51.2±7.3 years. There were 103 (60.2%) male and 68 (39.8%) female patients who comprised the study population; male to female ratio was reported to be 1.5:1. The duration of disease ranged from six to 18 months with a mean of 12.1±3.7 months. The disease was classified as Child-Pugh A in 20 (11.7%) patients, Child-Pugh B in 67 (39.2%) patients, and Child-Pugh C in 84 (49.1%) patients. Forty-four (25.7%) patients were hypertensive while 62 (36.3%) were diabetic. BMI of these patients ranged from 21.3 kg/m^2^ to 29.9 kg/m^2^ with a mean of 25.9±2.4 kg/m^2^ as shown in Table 1.

**Table 1:** Baseline characteristics of the study sample BMI- body mass index

Characteristics	Participants n=171
Age (years)	51.2±7.3
<45 years	20 (11.7%)
≥45 years	151 (88.3%)
Gender	
Male	103 (60.2%)
Female	68 (39.8%)
Duration of disease (months)	12.1±3.7
<1 year	76 (44.4%)
≥1 year	95 (55.6%)
Severity of disease	
Child-Pugh A	20 (11.7%)
Child-Pugh B	67 (39.2%)
Child-Pugh C	84 (49.1%)
Hypertension	
Yes	44 (25.7%)
No	127 (74.3%)
Diabetes	
Yes	62 (36.3%)
No	109 (63.7%)
BMI (kg/m^2^)	25.9±2.4
20-25 kg/m^2^	69 (40.4%)
25-30 kg/m^2^	102 (59.6%)

Serum LDL level varied from 44 mg/dl to 165 mg/dl with a mean of 76.95±26.10 mg/dl, while HDL varied from 28 mg/dl to 56 mg/dl with a mean of 39.26±5.93 mg/dl. Triglycerides levels were reported to vary from 79 mg/dl to 207 mg/dl with a mean of 100.24±26.88 mg/dl, and serum total cholesterol levels were recorded to vary from 104 mg/dl to 209 mg/dl with a mean of 147.82±28.67 mg/dl as shown in Table 2.

**Table 2:** Serum lipid profile of patients with chronic liver disease (n=171) LDL- low-density lipoprotein; HDL- high-density lipoprotein

Lipid profile	Serum level (mg/dl)
LDL	76.95±26.10
HDL	39.26±5.93
Total cholesterol	147.82±28.67
Triglycerides	100.24±26.88

There was no statistically significant difference in the mean serum values of LDL, HDL, triglycerides, and total cholesterol across various subgroups based on patient's age, gender, duration of disease, BMI, and diabetic and hypertensive status was reported. However, mean serum values of LDL (113.15±14.08 vs. 95.58±14.25 vs. 53.46±5.90 mg/dl; p-value 0.001), HDL (50.60±3.19 vs. 40.70±2.95 vs. 35.40±3.88 mg/dl; p-value 0.001), total cholesterol (174.20±17.33 vs. 164.00±17.82 vs. 128.64±24.73 mg/dl; p-value 0.001), and triglycerides (127.15±8.98 vs. 100.84±27.12 vs. 93.36±25.56 mg/dl; p-value 0.001) decreased significantly with increasing severity of disease as shown in Tables 3-6.

**Table 3:** Stratification of serum LDL level (mg/dl) across various subgroups of patients with chronic liver disease (n=171) LDL- low-density lipoprotein; BMI- body mass index

Subgroups	n	Serum LDL level (mean±SD)	P-value
Age (years)			
<45 years	20	75.45±24.68	0.786
≥45 years	151	77.15±26.36	
Gender			
Male	103	76.19±25.80	0.644
Female	68	78.09±26.72	
Duration of disease (months)			
<1 year	76	76.74±25.41	0.925
≥1 year	95	77.12±26.78	
Severity of disease			
Child-Pugh A	20	113.15±14.08	0.001
Child-Pugh B	67	95.58±14.25	
Child-Pugh C	84	53.46±5.90	
Hypertension			
Yes	44	77.36±27.63	0.903
No	127	76.80±25.67	
Diabetes			
Yes	62	74.90±30.56	0.442
No	109	78.11±23.27	
BMI (kg/m^2^)			
20-25 kg/m^2^	69	74.07±25.34	0.237
25-30 kg/m^2^	102	78.89±26.56	

**Table 4:** Stratification of serum HDL level (mg/dl) across various subgroups of patients with chronic liver disease (n=171) HBL- high-density lipoprotein; BMI- body mass index

Subgroups	n	Serum HDL level (mean±SD)	P-value
Age (years)			
<45 years	20	39.10±3.70	0.900
≥45 years	151	39.28±6.17	
Gender			
Male	103	38.94±5.84	0.393
Female	68	39.74±6.08	
Duration of disease (months)			
<1 year	76	39.58±6.03	0.527
≥1 year	95	39.00±5.86	
Severity of disease			
Child-Pugh A	20	50.60±3.19	0.001
Child-Pugh B	67	40.70±2.95	
Child-Pugh C	84	35.40±3.88	
Hypertension			
Yes	44	39.50±6.22	0.754
No	127	39.17±5.85	
Diabetes			
Yes	62	39.08±5.84	0.770
No	109	39.36±6.01	
BMI (kg/m^2^)			
20-25 kg/m^2^	69	39.91±5.39	0.235
25-30 kg/m^2^	102	38.81±6.26	

**Table 5:** Stratification of serum total cholesterol level (mg/dl) across various subgroups of patients with chronic liver disease (n=171) BMI- body mass index

Subgroups	n	Serum total cholesterol level (mean±SD)	P-value
Age (years)			
<45 years	20	146.35±30.62	0.807
≥45 years	151	148.02±28.51	
Gender			
Male	103	147.91±29.38	0.961
Female	68	147.69±27.78	
Duration of disease (months)			
<1 year	76	147.76±29.24	0.980
≥1 year	95	147.87±28.36	
Severity of disease			
Child-Pugh A	20	174.20±17.33	0.001
Child-Pugh B	67	164.00±17.82	
Child-Pugh C	84	128.64±24.73	
Hypertension			
Yes	44	148.52±26.59	0.852
No	127	147.58±29.46	
Diabetes			
Yes	62	144.47±27.95	0.249
No	109	149.73±29.03	
BMI (kg/m^2^)			
20-25 kg/m^2^	69	145.91±28.46	0.475
25-30 kg/m^2^	102	149.12±28.88	

**Table 6:** Stratification of serum triglycerides level (mg/dl) across various subgroups of patients with chronic liver disease (n=171) BMI- body mass index

Subgroups	n	Serum triglycerides level (mean±SD)	P-value
Age (years)			
<45 years	20	94.25±11.09	0.290
≥45 years	151	101.03±28.24	
Gender			
Male	103	100.81±27.34	0.736
Female	68	99.38±26.34	
Duration of disease (months)			
<1 year	76	101.45±27.84	0.601
≥1 year	95	99.27±26.18	
Severity of disease			
Child-Pugh A	20	127.15±8.98	0.001
Child-Pugh B	67	100.84±27.12	
Child-Pugh C	84	93.36±25.56	
Hypertension			
Yes	44	102.14±25.86	0.588
No	127	99.58±27.29	
Diabetes			
Yes	62	97.95±22.64	0.403
No	109	101.54±29.03	
BMI (kg/m^2^)			
20-25 kg/m^2^	69	100.10±29.05	0.956
25-30 kg/m^2^	102	100.33±25.45	

Nineteen (11.1%) patients had hyperlipidemia (serum values of two or more parameters above normal), while 152 (88.9%) patients had normal lipid profiles. Three (1.8%) patients had higher LDL, 77 (45.0%) patients had higher HDL, 19 (11.1%) patients had higher total cholesterol, and eight (4.7%) patients had higher triglyceride levels as shown in Table 7. Very high or low levels were not observed in any patient.

**Table 7:** Pattern of dyslipidemia in patients with chronic liver disease (n=171) HDL- high-density lipoprotein; LDL- low-density lipoprotein

Lipid profile	Participants n=171
LDL	
Normal	168 (98.2%)
High	3 (1.8%)
HDL	
Normal	94 (55.0%)
High	77 (45.0%)
Total cholesterol	
Normal	152 (88.9%)
High	19 (11.1%)
Triglycerides	
Normal	163 (95.3%)
High	8 (4.7%)
Hyperlipidemia	
Yes	19 (11.1%)
No	152 (88.9%)

A significant difference was not found in the frequency of hyperlipidemia across various subgroups based on the patient's gender, age, BMI, duration of disease, or diabetic and hypertensive status. The frequency of hyperlipidemia decreased with the increasing severity of the disease, however, a statistically significant p-value of less than 0.05 was not reached (p-value=0.192), as shown in Table 8.

**Table 8:** Stratification of hyperlipidemia across various subgroups of patients with chronic liver disease (n=171) BMI- body mass index

Subgroups	n	Hyperlipidemia n (%)	P-value
Age (years)			
<45 years	20	2 (10.0%)	0.866
≥45 years	151	17 (11.3%)	
Gender			
Male	103	12 (11.7%)	0.782
Female	68	7 (10.3%)	
Duration of disease (months)			
<1 year	76	9 (11.8%)	0.786
≥1 year	95	10 (10.5%)	
Severity of disease			
Child-Pugh A	20	4 (20.0%)	0.192
Child-Pugh B	67	9 (13.4%)	
Child-Pugh C	84	6 (7.1%)	
Hypertension			
Yes	44	5 (11.4%)	0.951
No	127	14 (11.0%)	
Diabetes			
Yes	62	7 (11.3%)	0.955
No	109	12 (11.0%)	
BMI (kg/m^2^)			
20-25 kg/m^2^	69	8 (11.6%)	0.869
25-30 kg/m^2^	102	11 (10.8%)	

## Discussion

Liver injury persisting six months or more is a manifestation of CLD, which can be caused by infection, inflammation, toxic, or congenital predisposition. Dyslipidemia is one of the systemic effects produced by CLD. Healing response due to liver injury causes hepatic fibrosis [9,10]. Liver impairment causes disturbed protein anabolism, excretion of bilirubin, and lipid metabolism. Such patients should be managed to improve liver metabolism [9]. Lipid profile monitoring is not a part of the routine management of such patients in our hospital; however, recent studies showed that lipid profile varies with the severity of disease and should be considered in the management of such patients [4,7].

Patients with CLD in our study had a mean age of 51.2±7.3 years. A similar mean age of 52±9 years was reported by Ali et al. (2008) in patients presenting with CLD at Muhammad Medical College Hospital, Mirpurkhas, while Hussain et al. (2014) reported it to be 51.12±6.03 years at Services Hospital, Lahore [11,12]. Achakzai et al. in 2016 and Almani et al. in 2008 also reported similar mean age of 54±11 years and 53.09±8.86 years respectively among such patients in their local population [13,14]. A similar mean age of 55.03±12.05 years has been reported by Mansour-Ghanaei et al. (2012) among Iranian such patients while Penteado et al. reported it to be 51.4±7.6 years in Brazil [15,16]. However, Bhattacharyya et al. (2016) and Deepika et al. (2015) had a much younger mean age of 45.8±10.45 years and 44±13.7 years respectively in Indian patients with CLD [17,18].

We observed a male to female ratio of 1.5:1 in our study. A similar male predominance among such patients has been reported previously by Ali et al. who observed it to be 1.5:1 in the local population [11]. El-Feki et al. in 2016 reported a similar male to female ratio of 1.5:1 among Egyptian patients with CLD [19]. Mansour-Ghanaei et al. in 2012 reported a male to female ratio of 1.9:1 among CLD patients in Iran [15]. Achakzai et al. in 2016 however observed a female predominance in CLD patients presenting at Dow University Hospital, Karachi with a male to female ratio of 1:1.5 [13].

In the present study, the disease was classified as Child-Pugh A in 20 (11.7%) patients, Child-Pugh B in 67 (39.2%) patients, and Child-Pugh C in 84 (49.1%) patients. Our observation is similar to that of Shaikh et al. (2011) who had a similar frequency of Child-Pugh Class-A (12.2%), Class-B (39.2%), and Class-C (48.6%) among cirrhotic patients at Liaquat University Hospital, Jamshoro [20]. While Naqvi et al. (2016) observed the frequency of Class-A, Class-B, and Class-C to be 11.4%, 31.4%, and 57.2%, respectively, among such patients at Dow University of Health Sciences, Karachi [21].

We observed that 25.7% of patients with CLD were hypertensive while 36.3% of patients were diabetic. Our results are similar to those of Arshad et al. (2016) who reported a similar frequency of 33.5% for diabetes among CLD patients presenting at Medical Unit III, Services Hospital, Lahore [22]. Similar diabetic rates have also been reported by Sigal et al. in 2006 (30.8%) in the American population, while Garcia-Compean et al. in 2009 reported the frequency of diabetic patients (30.0%) in Mexican patients with CLD [23, 24]. Unger et al. in 2009 reported a comparable frequency of 23.6% for hypertension in Australian patients [25].

We observed that the mean BMI of patients in our study was 25.9±2.4 kg/m^2^. Our observation is in line with that of Zou et al. (2016) who reported a similar mean BMI of 26.2±3.8 kg/m^2^ among Chinese patients with CLD [26].

The mean serum values of LDL, HDL, total cholesterol, and triglycerides decreased significantly with increasing severity of disease on Child-Pugh Classification as mentioned in Tables 3-6. Our observation is in line with a local study where Subhan et al. (2012) observed CLD patients at Khyber Teaching Hospital, Peshawar, and reported a similar significant decrease in serum LDL (110.6±29.5 vs. 96.3±49.2 vs. 56.5±26.6 mg/dl; p-value≤0.05), HDL (47.0±19.2 vs. 42.0±12.2 vs. 35.3±13.3 mg/dl; p-value≤0.05), total cholesterol (165.6±36.6 vs. 156.6±63.9 vs. 119.9±32.9 mg/dl; p-value≤0.05), and triglycerides (127.8±58.6 vs. 98.7±55.6 vs. 96.0±42.3 mg/dl; p-value≤0.05) levels with increasing severity of disease from Child-Pugh Class A vs Child-Pugh Class B vs Child-Pugh Class C [27].

Our observation is also in line with that of Ghadir et al. (2010) who observed CLD patients in Iran and reported similar remarkable decrease in serum LDL (107.6±29.5 vs. 96.4±58.5 vs. 59.5±28.9 mg/dl; p-value≤0.05), HDL (49.0±21.6 vs. 40.0±14.3 vs. 37.4±15.5 mg/dl; p-value≤0.05), total cholesterol (166.5±37.9 vs. 161.2±71.5 vs. 121.2±31.7 mg/dl; p-value≤0.05), and triglycerides (121.9±57.7 vs. 90.6±50.5 vs. 92.0±48.7 mg/dl; p-value≤0.05) levels with increasing severity of disease [28].

We observed that 11.1% of CLD patients had hyperlipidemia (serum values of two or more parameters above normal), while 88.9% of patients had normal lipid profile. Our observation is in line with that of Deeb et al. (2018) who had a similar frequency of 11.7% for hyperlipidemia in patients with CLD in the United Arab Emirates (UAE) [29]. Unger et al. in 2019 reported a comparable frequency of 13.0% for hyperlipidemia among Australian CLD patients [25]. Mehboob et al. in 2007 conducted a similar study at Sheikh Zayed Medical College, Rahim Yar Khan observed a relatively lower frequency of hyperlipidemia among CLD patients and reported it to be 7.5%, while a much higher frequency of 23.9% has been reported by Sohail et al. in 2020 at Bahawal Victoria Hospital, Bahawalpur [4,30].

A large sample size of 171 cases, strict exclusion criteria, and stratification of the results to minimize bias were the strengths of our study. However, a possible limitation could be our inability to account for serum lipid profile’s role in terms of response to treatment and mortality which could have highlighted its role in CLD patients and could have helped in the risk stratification and management of these patients.

## Conclusions

A remarkable proportion of CLD patients had hyperlipidemia which varied with the severity of disease on Child-Pugh classification necessitating routine monitoring of lipid profile of such patients for timely identification and management. This would improve the outcome of CLD patients. Our results suggest that lipid profile has an important role in the risk stratification and management planning of CLD patients and warrants further studies in this regard.
